# Label-Free Colorimetric Detection of Mercury (II) Ions Based on Gold Nanocatalysis

**DOI:** 10.3390/s18092807

**Published:** 2018-08-25

**Authors:** Pei-Chia Yang, Tsunghsueh Wu, Yang-Wei Lin

**Affiliations:** 1 Department of Chemistry, National Changhua University of Education, 1, Jin-De Road, Changhua City 50007, Taiwan; po840603@yahoo.com.tw; 2 Department of Chemistry, University of Wisconsin-Platteville, 1 University Plaza, Platteville, WI 53818-3099, USA

**Keywords:** colorimetric detection, mercury, HEPES-gold nanostar, catalytic reduction

## Abstract

Herein, a label-free colorimetric nanosensor for Hg(II) is developed utilizing the hindering effect of Hg(II) on the kinetic aspect of gold nanoparticle (AuNPs) growth on the surface of gold nanostars (AuNSs). H-AuNS probes are synthesized and modified by 2-[4-(2-hydroxyethel) piperazine-1-yl] ethanesulfonic acid (HEPES). After the formulation of the reagents and testing conditions are optimized, HEPES-capped AuNSs (H-AuNSs) demonstrates good selectivity and sensitivity towards Hg(II) determination. A H-AuNS probe, in the presence of HCl/Au(III)/H_2_O_2_, is capable of detecting a Hg(II) concentration range of 1.0 nM–100 µM, with a detection limit of 0.7 nM, at a signal-to-noise ratio of 3.0, and a visual detection limit of 10 nM with naked eyes. For practicality, the H-AuNS probe is evaluated by measuring Hg(II) in the environmental water matrices (lake water and seawater) by a standard addition and recovery study. The detection limits for environmental samples are found to be higher than the lab samples, but they are still within the maximum allowable Hg concentration in drinking water (10 nM) set by the US Environmental Protection Agency (EPA). To create a unique nanosensor, the competitive interaction between Hg(II) and Pt(IV) toward the H-AuNSs probe is developed into a logic gate, improving the specificity in the detection of Hg(II) ions in water samples.

## 1. Introduction

The environmental conservation of fresh water resources and the efforts to protect fresh water from hazardous contaminants have become a primary priority in many countries. Mercury (Hg), existing in nature and being constantly produced by industries, had posed serious risks to the environment and has already caused harm to humans from its toxicity [1,2,3,4]. Early detection and intervention of the suspected pollution are critical to prevent a potential environmental tragedy such as the Minamata Disaster in Japan in 1956. Early detection can succeed if a simple and sensitive measuring tool is developed to quantify Hg(II) in fresh water resources. At present, several methods have been used for Hg(II) determination, such as atomic absorption spectrometry (AAS), atomic fluorescence spectrometry (AFS), stripping voltammetry, and polarography [5,6,7]. These analytical methods offer advantages in terms of good stability, selectivity, sensitivity, and legal acceptability as the national standard detection methods for many countries. The drawbacks of these methods include bulky instrumentation, high cost per analysis, long analysis time, stringent requirements of sample storage, highly trained personnel, and relatively expensive instruments, which thereby limits their use for fast Hg(II) monitoring in the field. Hence, a portable, facile, rapid, and low cost colorimetric method for Hg(II) detection is needed.

Gold nanoparticles (AuNPs) have been exploited for chemical sensors and biosensors because of their unique physical and optical properties such as surface plasmon resonance (SPR), surface enhanced Raman scattering (SERS), and catalytic properties [8,9]. Currently, numerous detection methods of Hg(II) based on AuNPs have been reported, such as the aptamer and the functionalized AuNP nanosensor [10,11,12,13,14]. Much previous work utilized the aggregation of functionalized AuNPs from their chemical interaction with Hg(II) to produce a change in optical properties of AuNPs, with limit detections of 10 to 250 nM [8,9]. However, the success of Hg(II) induced aggregation is highly dependent on the recognized ligand, which greatly influences the size of nanoparticles [15]. The induced aggregate can also be influenced by the ionic strength of the sample matrix. Our previous study has demonstrated a sensitive and selective method for Hg(II) detection based on the silver nanoparticle catalytic assisted growth of AuNPs in the presence of Au(III)/HCl/H_2_O_2_ [16]. But the detection principle relies greatly on surface enhanced Raman scattering, which requires complex instrumentation and a tedious operation procedure as the main challenges for Raman based detection.

In this study, 2-[4-(2-hydroxyethel) piperazine-1-yl] ethanesulfonic acid (HEPES)-capped gold nanostars (H-AuNSs) were synthesized, and they showed a promising catalytic reduction of Au(III), which can be inhibited by the presence of Hg(II). The ultraviolet–visible spectroscopy (UV-Vis), transmission electron microscopy (TEM), and energy-dispersive X-ray spectroscopy (EDX) spectra were employed to elucidate its sensing mechanism. The goal of this study is to demonstrate a label-free, rapid, low-cost, highly selective and sensitive colorimetric method for detection of Hg(II) in natural water matrices. Although the use of HEPES for the synthesis of AuNPs has been reported during the course of the present investigation [17,18,19,20,21,22,23], the properties of catalytic reduction of Au(III) in the presence of Hg(II) have not been investigated. Compared to the commonly used chemicals (such as cetyltrimethylammonium bromide, sodium dodecyl sulfate, bis (p-sulfonatophenyl) phenylphosphine dipotassium salt, and poly(vinylpyrrolidone)) for the synthesis of AuNSs [24,25,26,27,28,29,30], HEPES is considered an environmentally-friendly green reagent and is used extensively in chemistry and biochemistry laboratories. This is because it possesses several properties, including maximum aqueous solubility, minimal salt and temperature effects, and high chemical stability. Finally, a NIMPLY gate is the negation of material implication [31]. This means that the material nonimplication from P to Q is true only if P is true and Q is false. Therefore, the competitive interactions between the Hg(II) and Pt(IV) toward the H-AuNSs allowed us to develop a NIMPLY logic gate to improve the specificity of this nanosensor.

## 2. Materials and Methods

### 2.1. Chemicals

All chemicals were purchased from Sigma-Aldrich (Milwaukee, WI, USA). All the reagents including HAuCl_4_, H_2_O_2_, HCl, NaOH, HEPES, HgCl_2_, CoCl_2_, Cu(NO_3_)_2_·2.5H_2_O, Ni(NO_3_)_2_·6H_2_O, H_2_PtCl_6_, ZnCl_2_, NaNO_3_, KNO_3_, Ba(NO_3_)_2_, Mg(NO_3_)_2_·6H_2_O, CaCl_2_·2H_2_O, Sr(NO_3_)_2_, FeCl_2_, FeCl_3_, Cd(NO_3_)_2_·4H_2_O, and Pb(NO_3_)_2_ were commercially available and of analytical reagent grade. Ultrapure water (18.2 MΩ-cm resistivity) was used from a Milli-Q ultrapure system (Millipore, MA, USA).

### 2.2. Preparation of H-AuNSs

H-AuNSs were synthesized by reducing Au(III) chloride in HEPES buffer at room temperature [18]. The H-AuNSs were prepared by mixing 100 μL of 0.4 mM HAuCl_4_ with 100 μL of 200 mM HEPES buffer. The mixed solution was shaken for 1 min and then left to grow in the dark for 24 h. For simplicity, the concentration of the as-prepared H-AuNS solution is defined as 1×.

### 2.3. Characterization

A JEOL 2010 TEM (JEOL, Tokyo, Japan) with the acceleration voltage of 200 kV was used to study the morphologies of the prepared H-AuNSs and AuNPs. EDX spectroscopy (Bruker Nano, Berlin, Germany) was used to reveal their elemental composition. An Evolution 200 UV-Vis spectrometer (ThermoFisher Scientific, Waltham, MA, USA) was used to record the UV-Vis spectra of the prepared H-AuNS and AuNP solutions. A dynamic light scattering (DLS) spectrophotometer (SZ-100, Horiba, Kyoto, Japan) was used to measure the hydrodynamic radius of the prepared H-AuNSs and AuNPs.

### 2.4. General Procedure for Sensing Hg^2+^ Ions

Stock solutions of the metal ions (0.1 M) were prepared in 0.1 M HNO_3_. For the selectivity study (*n* = 3), 100 μL aliquots of 150 μM metal ion solution and 15 μM Hg(II) were prepared and then separately mixed with 200 μL H-AuNS seed solution (1×) for 3 min. Next, the mixtures were added to 1.2-mL ultrapure water containing 150 μL HCl (10 mM), 100 μL HAuCl_4_ (0.1%), and 50 μL H_2_O_2_ (0.3%). The mixtures were equilibrated at 60 °C for 10 min. Finally, the UV-Vis spectra were measured using an Evolution 200 UV-Vis spectrometer. The formation of AuNPs could be kept at 4 °C and exhibited no precipitation for 3 days.

### 2.5. General Procedure for NIMPLY Logic Gate

For the NIMPLY logic gate study (*n* = 3), 100 μL aliquots of 150 μM Pt(IV) and 150 μM Hg(II) were simultaneously mixed with 200 μL H-AuNS seed solution (1×) for 3 min. Next, the mixtures were added to 1.1-mL ultrapure water containing 150 μL HCl (10 mM), 100 μL HAuCl_4_ (0.1%), and 50 μL H_2_O_2_ (0.3%). The mixtures were equilibrated at 60 °C for 10 min. Finally, the UV-Vis spectra were measured using an Evolution 200 UV-Vis spectrometer.

## 3. Results and Discussion

### 3.1. Sensing Mechanism

**Scheme 1:**
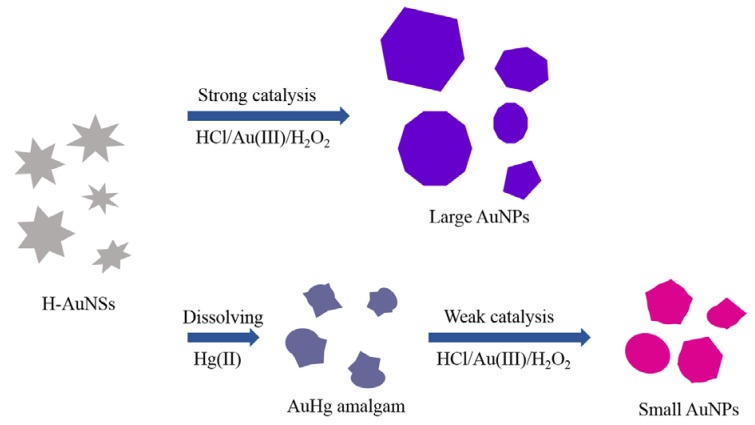
Colorimetric detection of Hg(II) based on its inhibition of H-AuNS catalytic reduction of Au(III) in the presence of HCl and H_2_O_2_.

The sensing mechanism for this study is illustrated in Scheme 1. H-AuNSs were prepared by the reduction of gold chloride in a HEPES buffer at room temperature [18]. Upon the addition of Au(III) to H-AuNSs solution, Au(III) ions were adsorbed onto the H-AuNS surface from electrostatic interactions between Au(III) and the negatively charged HEPES on the H-AuNS surface [32]. Subsequently, these Au(III) ions were reduced to Au(I) due to the reducing power of the HEPES layer. As a result, the standard electrode potential of Au(I) (E^0^_Au(I)/Au_: 1.83 V) is larger than that of H_2_O_2_ (E^0^_H2O2/H2O_: 1.77 V), favoring the reduction at the solid–liquid interface to form AuNPs in the presence of HCl [33]. When Hg(II) ions were pre-mixed with H-AuNSs, the formation of a AuHg amalgam occurred [34,35] and reduced the H-AuNSs surface area for adsorption of Au(III) on the gold surface. The un-reacted H-AuNSs and the AuHg amalgam acted as seeds for the growth of AuNPs in the HCl/Au(III)/H_2_O_2_ solution. As a result, the color of the H-AuNS solution gradually turned from light purple to red during this process. The color intensity of a treated water sample offers the visual determination of mercury, and it can directly relate to the amount of Hg(II) present in the water samples. Furthermore, the relative change of the SPR band in the resulting AuNPs, formed between the absence and presence of Hg(II) ions, can offer a more sensitive instrumental method to quantify Hg(II) in the water samples.

**Figure 1:**
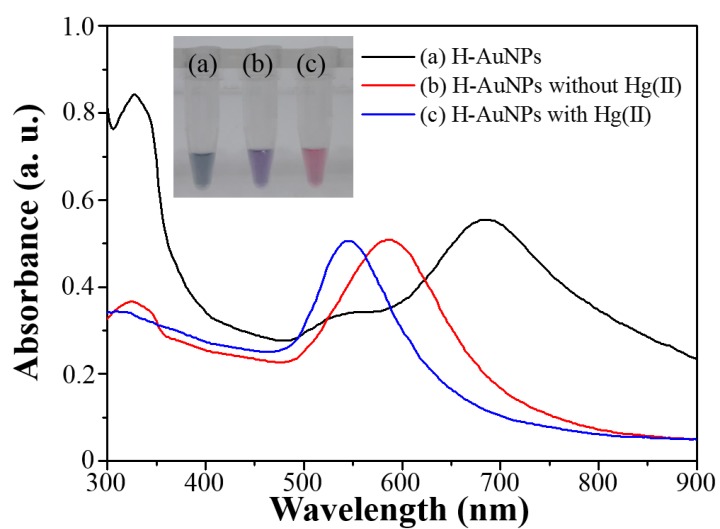
UV-Vis spectra of (**a**) the H-AuNS probe prior to the reactions and the H-AuNS probe, (**b**) without and (**c**) with Hg(II) (1.0 μM) in the presence of the HCl/Au(III)/H_2_O_2_ solution. Inset: color visualization for (a), (b) and (c).

Figure 1 shows the absorbance spectra of the H-AuNS probes without and with Hg(II) in the presence of the HCl/Au(III)/H_2_O_2_ solution. As can be seen in Figure 1 (black curve), the initial H-AgNSs possessed a SPR peak at 700 nm. This SPR band blue-shifted to 580 nm when 150 μL HCl (10 mM), 100 μL Au(III) (0.1%), and 50 μL H_2_O_2_ (0.3%) were added to the H-AuNS solution (red curve). After adding the HCl/Au(III)/H_2_O_2_ solution to the H-AuNS solution, a reduction reaction took place on the surface of H-AuNSs, generating a different morphology of AuNPs and causing the blue shift in the SPR band. After adding 100-μM Hg(II) to the H-AuNS solution and then following with the addition of HCl/Au(III)/H_2_O_2_ solution, the SPR absorbance further blue-shifted to 530 nm (blue curve), indicating the formation of small-sized AuNPs. We suggested that the AuHg amalgam acted as nucleation seeds for the growth of small AuNPs. Thus, the blue-shifted wavelength of the SPR band was due to both the change of the particle morphologies and the dielectric constants. This also explains why the color of the AuNP solution gradually became red, providing the sensing principle for the determination of Hg(II) in the aqueous solution. Therefore, the Hg(II) concentration can be determined by monitoring relative changes in the SPR band ratio ($\left(R-R_{0}\right)/R_{0}$) of the produced AuNPs, where R and R_0_ represent the SPR band ratio ($A_{580}/A_{530}$) of the AuNPs in the presence and absence of Hg(II), respectively. The higher relative changes in the SPR band ratio indicated a higher extent of the AuHg amalgam formation, resulting in a larger increase in the number of small AuNPs.

**Figure 2:**
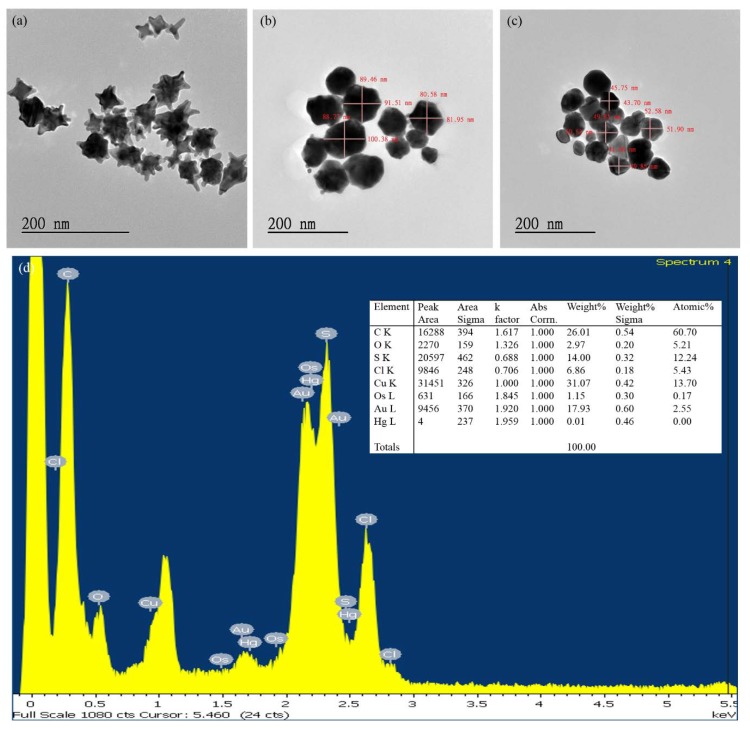
TEM images of (**a**) the H-AuNS probe prior to the reaction; and the H-AuNS probe (**b**) without and (**c**) with Hg(II) (1.0 μM) in the presence of HCl/Au(III)/H_2_O_2_ solution. (**d**) EDX spectrum of the H-AuNS probe with Hg(II) in the presence of HCl/Au(III)/H_2_O_2_ solution.

Figure 2 represents the TEM images and EDX spectrum of the corresponding Au nanomaterials. Prior to the reaction, the H-AgNSs typically had 4–8 branches, with an average tip-to-tip distance of 42.5 ± 0.7 nm from TEM measurements (Figure 2a). In the absence of Hg(II), Au(III) adsorbed on the H-AuNS surface and reduced to form irregular AuNPs with a diameter of 88.8 ± 6.5 nm (Figure 2b). However, when Hg(II) was present, the AuNPs produced were smaller than those without Hg(II) (Figure 2c), and they possessed more spherical morphology with an average diameter of 48.2 ± 4.0 nm. These observations reveal that Hg(II) caused the shape and size change of H-AuNSs in the HCl/Au(III)/H_2_O_2_ system and enhanced the formation of a thermodynamically-favored resembling spherical surface. Meanwhile, EDX analysis provided more evidence that AuNSs and AuNPs for Figure 2a,b respectively contain no mercury element. The EDX spectrum of the produced AuNPs for Figure 2c indicates the co-existence of Au and Hg elements, supporting the formation of the AuHg amalgam (Figure 2d).

**Figure 3:**
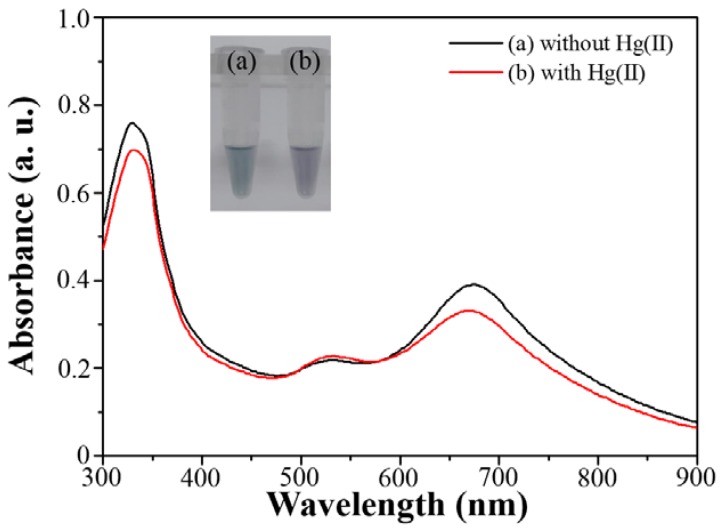
UV-Vis spectra of the H-AuNS probe (**a**) without and (**b**) with Hg(II) (1.0 μM) in the absence of HCl/Au(III)/H_2_O_2_ solutions. Inset: photographs of the prepared AuNPs solutions.

**Figure 4:**
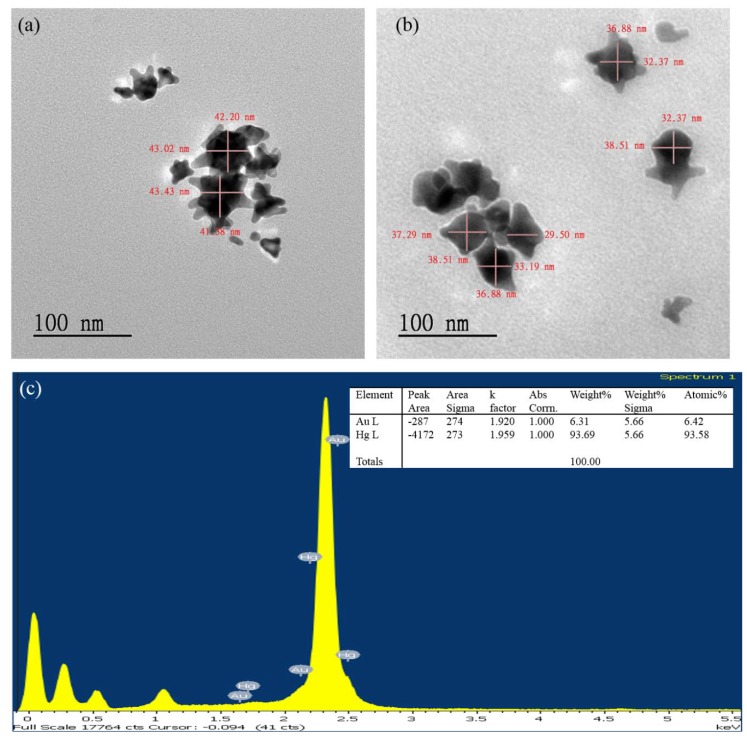
TEM images of the H-AuNS probe (**a**) without and (**b**) with Hg(II) (1.0 μM) in the absence of HCl/Au(III)/H_2_O_2_ solutions; (**c**) EDX spectrum of the H-AuNS probe with Hg(II) in the absence of HCl/Au(III)/H_2_O_2_ solution.

**Figure 5:**
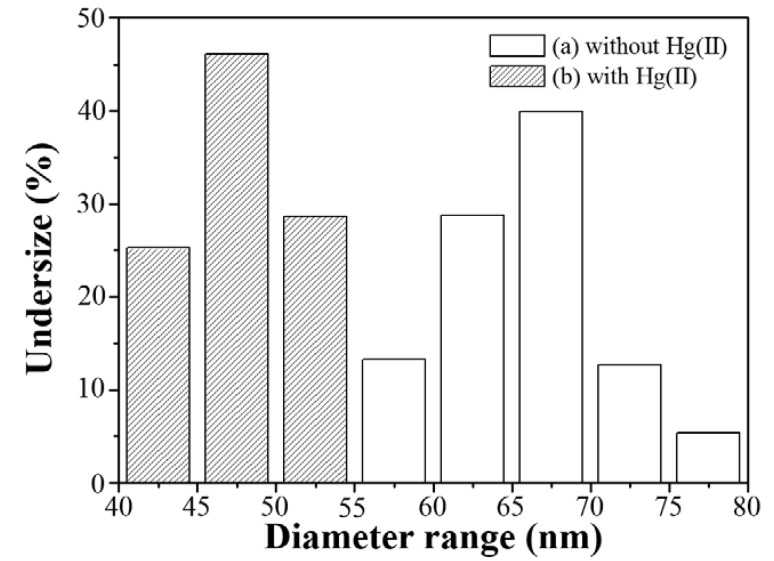
Hydrodynamic diameter distribution of the AuNPs solutions (**a**) without and (**b**) with Hg(II) (1.0 μM) by dynamic light scattering technique.

Investigation on the effects of Hg(II) on the size and shape change in H-AsNSs was carried out with UV-Vis, TEM, and dynamic light scattering to gain a deeper understanding of the H-AuNS dissolution process. First, the H-AuNS probe was mixed with Hg(II) alone, and the change in its optical property from UV-Vis was observed in Figure 3. Adding Hg(II) to the H-AuNSs solution decreased the SPR absorbance (red curve), which reflects the change of the particle morphologies and dielectric constants upon the reaction between AuNSs and Hg(II). Second, the morphology change from the reaction between AuNS and Hg(II) was observed by TEM, as shown in Figure 4. From the TEM image, noticeable branches were observed in AuNSs, and the number and the size of branches were reduced after reacting with Hg(II) for 10 min. The size was reduced upon the addition of Hg(II); the widths of the H-AuNSs without and with Hg(II) were 42.5 ± 0.7 and 38.4 ± 3.3 nm, respectively. Third, EDX again confirmed the coexistence of Au and Hg elements, as shown in Figure 4b. Finally, dynamic light scattering determined that the effective hydrodynamic diameter of the AuNPs without and with Hg(II) were 67.5 ± 8.8 and 48.3 ± 4.4 nm, respectively (Figure 5). All results indicated that Hg(II) effectively dissolved H-AuNSs because of the AuHg amalgam formation.

### 3.2. Sensing System Optimization

**Figure 6:**
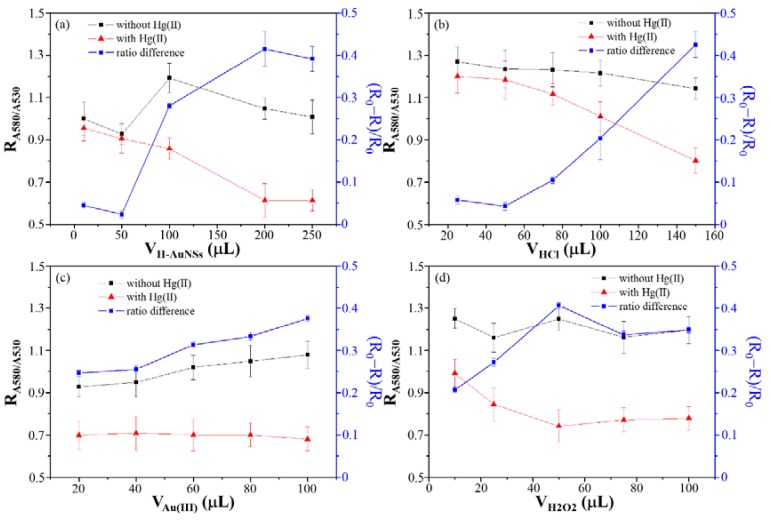
SPR band ratios (R: A_580_/A_530_) to evaluate the catalytic effects of the H-AuNS probe (■) on AuNP growth without (■) and with (▲) of Hg(II) (1.0 μM) in the presence of HCl/Au(III)/H_2_O_2_ solution, at different volumes of (**a**) H-AuNSs, (**b**) HCl, (**c**) Au(III), and (**d**) H_2_O_2_. Error bars represent standard deviations from three repeated experiments.

Factors for the performance of the H-AuNS probe in Hg(II) determination, such as solution volumes of reagents (1× H-AuNSs, 10 mMHCl, 0.1% Au(III), and 0.3% H_2_O_2_), reaction temperature, and time, were evaluated based on the relative change in the SPR band ratio, $\left(R_{0}-R\right)/R_{0}$, of the AuNPs, where R and R_0_ represent that SPR band ratio (A_580_/A_530_) of the AuNPs in the presence and absence of Hg(II) ions, respectively. The volume of reagents was studied first to generate an optimized formulation for the subsequent study and real sample analysis. The effect of H-AuNS volumes on the determination of Hg(II) (1.0 μM) was investigated in the range of 10 μL to 250 μL, and Figure 6a shows that a volume of 200 μL offers the highest SPR band ratio. A higher concentration of H-AuNSs provides a seeding surface to form AuNPs subsequently in the presence of HCl/Au(III)/H_2_O_2_. In the presence of Hg(II), the formation of AuNPs was hindered by the reduced AuNS surface area and the possible change on surface composition, resulting in favorable formation of small AuNPs. Next, the role of HCl in this sensing platform is to catalyze the formation of AuNPs in the presence of the Au(III)/H_2_O_2_ solution. 150 μL HCl was determined as the optimum volume (Figure 6b). With the set H-AuNS and HCl volume, the optimum volume of Au(III) for the further growth AuNPs was found to be 100 μL (Figure 6c). Moreover, the dissolving effect gradually increased with an increasing volume of H_2_O_2_ before reaching a plateau at about 50 μL of H_2_O_2_ added (Figure 6d). Thus, the optimum volume of H_2_O_2_ was 50 μL for the following experiment. Interestingly, the optimization study has shown that the sensitivity of the H-AuNS probe increases with an increase in all four reagents, as they all favor the formation of small AuNPs in the presence of Hg(II), supporting the mechanism of sensing strategy.

**Figure 7:**
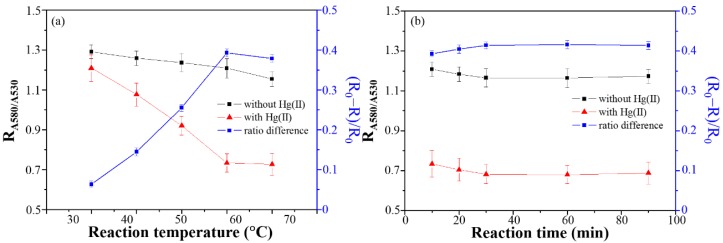
Absorbance ratios (R: A_580_/A_530_) and catalytic effects (■) for the H-AuNS probe without (■) and with (▲) of Hg(II) (1.0 μM) in the presence of HCl/Au(III)/H_2_O_2_ solution, at different (**a**) reaction temperatures and (**b**) times. Error bars represent standard deviations from three repeated experiments.

The effect of temperature on the change in SPR band ratio was investigated over the range 30–70 °C. In Figure 7a, the SPR band change ratio in the sensing system increased with an increase in the reaction temperature before reaching a plateau at 60 °C (Figure 7a). As the temperature increased, the frequency of collisions between Hg(II) and H-AuNSs increased, resulting in an increased degree of dissolution for H-AuNSs. At a fixed temperature at 60 °C, the change in SPR ratio only increased slightly as time elapsed, reaching a maximum at 30 min, which was attributed to the dissolving rate for the H-AuNSs reaching the maximum (Figure 7b). Fortunately, a majority of AuNPs was formed and stabilized within the first 10 min of reaction. Therefore, a temperature of 60 °C and a reaction time of 10 min were chosen for water analysis.

### 3.3. Selectivity and Sensitivity of the Sensing System

**Figure 8:**
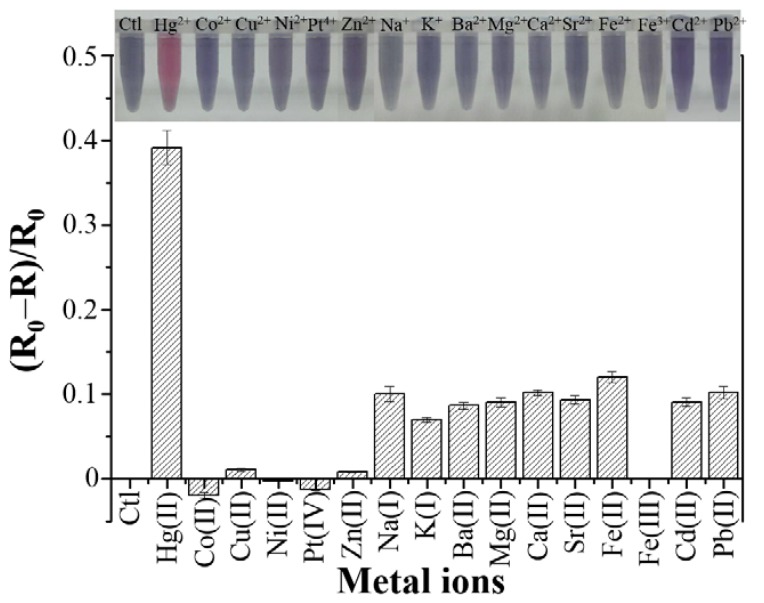
Selectivity of the H-AuNS probe in the presence of HCl/Au(III)/H_2_O_2_ solution toward Hg(II) ions. The Hg(II) concentration was 1.0 μM, and the concentration of each of the other metal ions was 10 μM. Inset: photographs of the H-AuNS probe with different metal ions in the presence of HCl/Au(III)/H_2_O_2_ solution. Error bars represent standard deviations from three repeated experiments.

With the optimized conditions, the selectivity of the H-AuNS probe was evaluated in the presence of other metal ions at a concentration of 10.0 μM. As shown in Figure 8, the change in the SPR ratio by non-mercuric metal ions was at most insignificant compared with the system with 1.0 μM Hg(II) alone. On the basis of analysis of variance (95% confidence level, F_critical_ = 1.66) and the least significant difference method, the $\left(R_{0}-R\right)/R_{0}$ value obtained using the H-AuNs probe in the presence of Hg(II) is a significant difference among those in the presence of non-mercuric metal ions. This is because Hg(II) effectively dissolved H-AuNSs due to the AuHg amalgam formation, resulting in the growth of small AuNPs. Among all the ions tested, iron (II), sodium, calcium, lead, and cadmium also showed some degree of change. This suggests these ions, at a higher concentration, can pose some challenges in water analysis, but standard addition, shown in a later section of this report, was developed to reduce their interferences.

**Figure 9:**
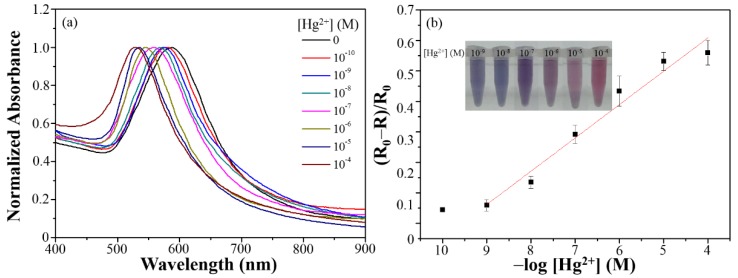
(**a**) UV–Vis absorbance spectra and (**b**) linear responses of the catalytic effect plotted against the Hg(II) concentration. Inset: photographs of the H-AuNS probe in the presence of HCl/Au(III)/H_2_O_2_ solution at different Hg(II) concentrations. Error bars represent standard deviations from three repeated experiments.

The sensitivity of the H-AuNS probe in the presence of the HCl/Au(III)/H_2_O_2_ solution was studied at its optimum conditions after being equilibrated at 60 °C for 10 min by lowering the Hg(II) concentration to a level where the color change of the AuNP solution was detectable with the naked eye. At the same time, the UV–Vis spectra of the AuNP solutions containing different Hg(II) concentrations were examined for the construction of a quantitative assay. A blue shift from the SPR perspective, or a red shift from the visual inspection, was distinctively observable with an increase in the Hg(II) concentration, as shown in Figure 9a. The color of the solutions changed from light purple to red as the Hg(II) concentration was increased from 1.0 nM to 100 μM (Inset image in Figure 9b). The visual detection limit for Hg(II) was 10.0 nM with the naked eye. A linear relationship was found between the ratio $\left(R_{0}-R\right)/R_{0}$ and the logarithmic concentration of Hg(II) over the range 1.0 nM–100 μM, and the linear correlation coefficient was 0.9607. Considering a signal-to-noise ratio of 3, the limit of detection (LOD) for Hg(II) by the H-AuNS probe was 0.7 nM. This result suggested that the H-AuNS probe, in the presence of the HCl/Au(III)/H_2_O_2_ solution, is well-suited for monitoring Hg(II) ions in environmental water samples, as the probe can detect Hg(II) lower than US EPA drinking water standard for the highest Hg(II) concentration permitted in drinking water at 10.0 nM.

### 3.4. Application of the Sensing System

For practicality, the prepared H-AuNS probe in the presence of the HCl/Au(III)/H_2_O_2_ solution was used for detecting Hg(II) in environmental water matrices. A fresh water sample from Sun Moon Lake in Nantou and a seawater sample from the beach in Taichung were each filtered through a 0.2 μm membrane. The filtered samples were analyzed by ICP-MS. Both water samples contained Hg(II) concentrations below the LOD of our probe when 0.9 mL of the sample was added to 0.6 mL of HCl/Au(III)/H_2_O_2_ solution containing H-AuNS probes. Standard addition is a well-accepted quantitative analysis technique to analyze Hg(II) ions in complicated matrices. In this standard addition study, a linear correlation between the $\left(R_{0}-R\right)/R_{0}$ value and the spiked Hg(II) concentration was found over the Hg (II) concentration range of 10.0 nM–100.0 μM, and the recoveries for the lake water and seawater samples were determined to be 92.1–117.1% and 88.9–119.1%, respectively. The LODs for Hg(II) in the lake water and seawater samples were 6.3 and 8.9 nM, respectively, which are low enough for the H-AuNS probe to be promising in environmental monitoring.

**Figure 10:**
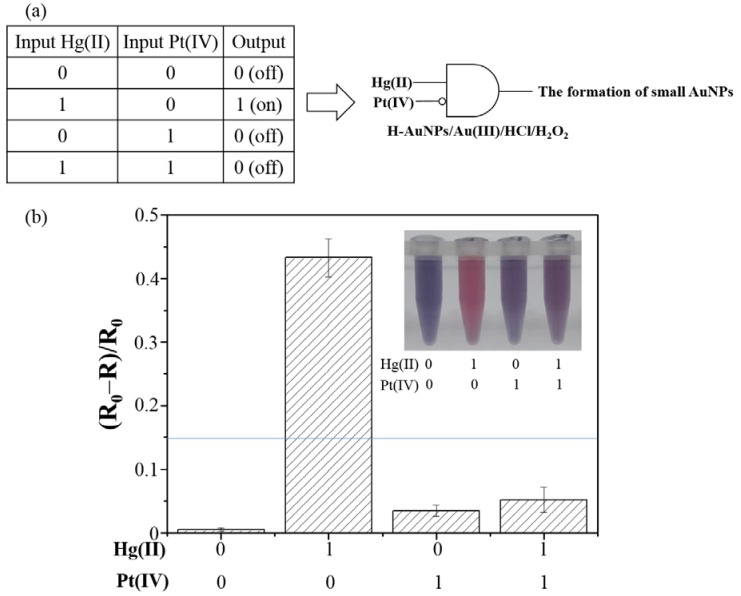
NIMPLY logic gate constructed using Hg(II)/Pt(IV) ions and the H-AuNS probe in the presence of HCl/Au(III)/H_2_O_2_ solution. (**a**) Schematic representation of the NIMPLY logic gate “Hg(II)/Pt(IV)(NIMPLY)–HAuNP”; (**b**) Bar diagram of output signals (formation of small AuNPs) in response to four different combinations of two inputs, Hg(II) (0, 10 μM) and Pt(IV) (0, 10 μM). The threshold line is set to the 0.3-fold signal output of true (on). Error bars represent standard deviations from three repeated experiments. Other conditions were the same as those described in Figure 1.

Construction of a NIMPLY logic gate with a H-AuNS probe was investigated to improve the specificity of Hg(II) detection. This unique logic gate used Hg(II) and Pt(IV) ions as input and the formation of small AuNPs as the output, as shown in Figure 10. A high output of signal coming from a high relative change in the SPR ratio or from a distinctive visual color change to red was assigned as the “ON” signal and output value of 1. This two-input NIMPLY logic gate produced a high output (output = 1) in the presence of a Hg(II) input alone (input for Hg(II) = 1, input for Pt(IV) = 0). But when only Pt(IV) was present (0, 1) and both Pt(IV) and Hg (II) inputs (1, 1) were present, the formation of small AuNPs was suppressed (output = 0) due to the preferential formation of Au/Pt alloy on the H-AuNS surface [36,37]. The nanoparticles after the logic gate experiment were collected and further analyzed by ICP-MS. The ICP-MS results show that the AuNP possessed 4055 ± 120 Hg atoms per AuNP and 3588 ± 100 Pt atoms per AuNP in the presence of Hg(II) and Pt(IV), respectively. But the number of Hg atoms were reduced dramatically when the H-AuNS probe was exposed to the mixture of Hg(II) and Pt(IV) with 388 ± 108 Hg atoms and 3248 ± 32 Pt atoms per AuNP. This study reveals that Pt(IV) forms an alloy with Au, which protects H-AuNSs and inhibits the formation of small AuNPs. When both Pt(IV) and Hg(II) are present (input = 1, 1) in the water sample, a stronger interaction between Pt(IV) and H-AuNSs (E^0^_Pt(IV)/Pt_: 1.48 V) compared to Hg(II) and H-AuNSs (E^0^_Hg(II)/Hg_: 0.85 V) takes place, significantly lowering the effect of Hg in dissolving branches of H-AuNSs and strongly hindering the formation of small-sized AuNPs(output = 0).

## 4. Conclusions

In summary, a label-free nanosensor (H-AuNSs) was developed for colorimetric detection of Hg(II). Based on dissolution of AuNSs in the presence of Hg(II) and formation of amalgam, the formation of AuNPs from HCl/Au(III)/H_2_O_2_ was hindered by the reduced effective surface area and elemental composition. In other words, the Hg(II) regulates the AuNP formation on the surface of H-AuNS and affects the shape and size of the resulting AuNPs significantly enough to cause a visible color change as low as 10 nM of Hg(II). Under the optimized conditions, the probe is selective to Hg(II) compared with other metal ions, producing a significant difference in the change of the SPR ratio, even at a low concentration of Hg(II) (1.0 μM). The linear detection range and LOD for Hg(II) in laboratory standards were 1.0 nM–100 μM and 0.7 nM, respectively. The good selectivity and sensitivity of this H-AuNSs probe offer portable detection of Hg(II) in environmental water matrices to enhance crucial environmental conservation efforts. Furthermore, the competitive interaction between Hg(II) and Pt(IV) toward the H-AuNSs probe enables the development of a NIMPLY logic gate through the regulation of the nanocatalytic reaction of the H-AuNSs probe, increasing the specificity of this H-AuNS-based Hg(II) assay.
